# On the Neck as a Medical Region; in Syncopal Seizure, &c.

**Published:** 1850-01

**Authors:** Marshall Hall


### PATHOLOGY AND PRACTICE OF MEDICINE.

### *On the Neck as a Medical Region ; in Syncopal Seizures ; &c.*

BY MARSHALL HALL, M. D., F. R. S., &c.

(Concluded.)

I trust the time will arrive when a minute knowledge of anatomy and physiology will be esteemed to be as essential to the physician as to the surgeon, and that this view of the subject will prevail, not in the profession only, but in the public. Then, and not till then, will the hydra-headed monster, quackery, become extinct. And yet it does not appear to be exacting too much to require, that he who undertakes to repair a complicated machine should first become acquainted with its structure and springs of action, and the nature of its varied derangements.

A useful work might be written on Medical Anatomy and Physiology. I have ventured to offer specimens of such a work, in treating of *the Neck* as a medical region, and *arrière circulation*. In the present paper I propose to add another topic to those already mentioned, in noticing briefly the effect of conditions *within* the cranium and spinal canal in inducing shock of the medulla oblongata, and of this on the heart and stomach.

It will be observed, that all kinds of seizure to which I have adverted, are *spinal* in their origin—emotion and causes of reflex action acting variously on this centre of the nervous system—whatever their *forms*, and whether these be cerebral or spinal, or, as I now proceed to describe them, cardiac or stomachic.

For the seizure is not always one affecting the intellect, or the muscular system ; sometimes on the contrary, there are pallor and faintishness, if not actual syncope, and nausea, if not vomiting.

This form of seizure—I purposely avoid the term epilepsy—arises from the same causes as the others to which I have adverted. They belong to a *Class* of affections, which, however various and varied in other respects, combine the symptoms of faintness or sickishness. A fall, or blow on the head ; an apoplectic seizure ; hydrencephalus ; loss of blood ; disgust, fear, and other forms of emotion, and sea-sickness, are of this kind.

A fall or blow on the head may be regarded as likely to be more or less serious in its ulterior effects, according as it is or is not followed by pallor and sickness. The apoplectic seizure is frequently marked by pallor and vomiting; a frequent, sometimes the *only*, symptom of incipient hydrencephalus, is vomiting. Every one knows the effect of certain emotions in blanching the cheeks, and inducing nausea; the effects of loss of blood and of posture during bloodletting or hæmorrhage ; the phenomena of sea-sickness : in all these cases it is probable that the *medulla oblongata* is the precise part of the nervous centres specially affected. It is on this part that the blow, or *contre-coup* in the case of accident, that certain emotions, that the diminished pressure of the column of spinal blood in bloodletting in the erect position, and that the varied pressure of that column on the sea, have their chief influence.

I have suggested the anatomy of the veins and muscles of the neck as an important subject of inquiry to the physician. I would now suggest a careful examination of the veins within the spinal canal and cranium, as presenting another topic in *medical anatomy* of great interest. Again, I would express my hope, that medicine, like surgery will one day be based on the sciences of anatomy and physiology, to the exclusion of ignorance and empiricism together. It was written on the portal of the school of PLATO ; Ουδεις αγεωμετρητος εισετω. Is it too much to ask of the legislature to enact that no one ignorant of anatomy and physiology shall prescribe or administer medicine, and cause the words Ουδεις ΑΦΥΣΙΟΛΟΓΟΣ εισιτω to be inscribed over our Medical Colleges ? Anatomy, physiology, DIAGNOSIS, pathology, (that is, living pathology,) therapeutics, (or the *modus operandi* of medicine),—such are the steps to be taken by the scientific physician in his visits to his patients. In the absence of any part of this knowledge, he may descend to what is termed experience or experiment. He must descend low, indeed, if he would place himself on a level with public expectation and opinion. Men in general have no higher ideas of medicine than those of a remedy, or of a collection of remedies, for a disease or diseases. Of pathology and of therapeutics, of the nature of morbid actions, and of the actions of remedies, and so of the Science and rational treatment of disease, no adequate idea exists in the public mind. But I proceed with my subject.

We do not find the most violent voluntary efforts to affect the brain. No delirium or loss of consciousness attends or follows them. There must be something *more* in *convulsive* actions than in mere *voluntary* efforts, however violent, to produce these effects. What is the nature of this additional impulse ? I imagine that the crushing influence of convulsive action not only compresses the *veins*, but extends even to the *arteries ;* and that the violence of action is thus applied in its unmitigated form to the *intermediate* capillary vessels ! On the face, especially in the eyelids and adjacent portions of integument, we observe minute ecchymoses, as the result of the epileptic convulsion, for instance ; how much more may it be supposed that the delicate tissues of the cerebrum and medulla suffer. We no longer wonder that delirium, or oblivion or unconsciousness, either momentary or more persistent—that a paroxysm of mania, a continued state of coma, that paralysis, or general convulsion should be the occasional result, the intermediate vessels being either gorged and distended with blood, or even ruptured, with its effusion—cerebral or medullary ecchymosis, or hæmorrhagy!

The only other circumstances in which I have seen the minute ecchymoses round the eye, to which I have adverted, are, parturition and violent retching and vomiting. The former actually induces, occasionally, a fit of apoplexy, or of mania. That the latter does not do so is astonishing, and owing perhaps to the simultaneous feebleness of the action of the heart, and emptied condition of the stomach, with the yielding of the closed glottis, and expiration.

The violent cough of pertussis sometimes issues in convulsion.

But the difference between the effects of all these violent efforts, and the condition induced by the simultaneous compression of the veins and arteries of the neck, *upwards*, will be obvious on the least reflection.

It is usual to observe in intense blushing, not only the face, but even the neck and bosom, involved in the suffusion. The course of the blood, not only of the external jugular, but of the external mammary vein, is arrested, the subclavian being compressed by spasm of the subclavius.

In violent and general convulsion, the whole surface of the body, the hands and the feet, became affected with lividity—an effect induced by closure of the larynx and violent expiratory efforts, with the continued and violent contraction of the muscles of the limbs on the deeply seated veins.

Disease of the heart induces similar effects on the *arrière* circulation more slowly, with congestion, or with effusion of blood or serum, in the lungs, liver, kidney, and in the hæmorrhoidal veins, as well as those of the head, neck, and extremities.

A contracted condition of the right side of the heart, especially such as would distend the vessels and induce swelling of the neck, would, I imagine, arrest the coronary circulation, and prove as it does probably, in too many instances, the cause of *sudden death*.

I may here remark that a painful paroxysmal spasm sometimes affects the muscles of the upper part of the thorax. I have seen two cases of this kind. I have not seen it described in any medical work.

There is something quite *special*, both in regard to the *cause* and *form* of seizures, and to the *susceptibility* to their return.

Infantile convulsions, arising, as they do, from causes peculiar to this age, do not present the protrusion of the tongue and its compression by the closure of the maxillæ, so usual in the adult; nor do they leave the patient subject to epileptic seizures in after life, so frequently as we might have expected.

A similar remark may be made in regard to puerperal convulsions; the epileptic patient is not specially liable to puerperal convulsions, nor do these leave the patient specially liable to epileptic seizures.

In epileptic patients in general, the cause and the form are usually the same in the same individual, and the susceptibility is a susceptibility to the same *kind* of seizure.

An unwholesome meal is frequently the common cause of the epileptic seizure in the *same* patient. In one patient there were regularly epileptic seizures at each catamenial period for more than ten years. No other excitant seemed to have any influence in inducing the paroxysms, the first of which occurred on the very *first* appearance of the catamenia. The rest occurred at each period only. The patient married at the age of eighteen, and was never pregnant.

The *form* of the malady is also usually similar in its return in the same patient; for instance, it is always cerebral, or always spinal, and, in the same case, similar in *form*.

I formerly objected to the use of the term epilepsy altogether, on account of the formidable meaning of this term, as generally used. For, in fact, the term designates two kinds of malady—one the most formidable and incurable with which we meet in practice—the other, by no means incurable. The term epilepsy, if not discarded, may be applied more extensively than is usually done to designate certain cases of sickness and syncope, greatly allied to the less formidable epilepsy; and in this manner the force of the term may be diminished. "Sick-headache," sickness, faintishness, vertigo, clammy perspiration, sickness the effect of disgust, are not dissimilar from the milder epilepsy—the "*petit mal*" of the French writers. The effect on the susceptible medulla oblongata of a swing, of sea-sickness, belong to the same *class* of morbid affections, and deserve the designation of μικρα επιληψια far more than that physiological act to which it had been applied, whilst the state of sleep may be viewed as a μικρα αποπληξια, induced by such a tonic action in the muscles of the neck, as, in the orbicularis, closes the eyelid.

There are seizures of a character altogether peculiar, and which combine, in the same patient, pallor, or pale lividity, faintness or actual syncope, sickishness or actual vomiting, sometimes with a clammy perspiration; or one or two of these symptoms may occur without the rest.

It is worthy of remark that the emotions, whilst each produces its own peculiar effect through the spinal and ganglionic systems, have their analogues, as it were, amongst the seizures of which the nervous system is the seat. Thus anger causes the countenance to flush or to turn pale, in various cases, and sometimes issues in apoplexy, epilepsy or syncope. Seizures, as I would use the term, induce precisely similar effects in different cases or circumstances. Even a fall or blow on the head, and the apoplectic seizure, is apt to be attended or followed by pallor and by vomiting, by which symptoms, indeed, its degree of severity is indicated.

The most minute inquiries ought to be made, in every case of sudden seizure, as to the appearance of the countenance. Pallor, and syncope, and sickness, probably depend on shock to the medulla oblongata. They become diagnostic, both of the seat and of the severity of the affection.

Another form of seizure is that of *delirium*. This may last an hour, several hours, or longer; it may even be protracted into a paroxysm of *mania*.

Previous to the delirium, which lasted in one case from three o'clock in the morning until six, pallor of the countenance followed by flushing was observed. After this paroxysm had passed off, the mind was observed to be enfeebled.

It was a *cardiac* or syncopal seizure followed by *cerebral* excitement.

The condition of the medulla oblongata has a special influence, through the medium of the pneumogastric nerve, on that of the heart.

Even in the batrachian, it is almost impossible to retain the circulation, after having destroyed or removed the medulla oblongata. In order to accomplish this in the frog, the brain must be removed first; then, after an interval of several hours, the lower part of the spinal marrow; and lastly, after another similar interval, the medulla oblongata. Even in this manner, it is only under favourable circumstances that the capillary circulation remains good.

We need not wonder that, in the higher orders of animals, and especially in the human subject, any affection of the medulla oblongata induces peculiar effects on the heart, and indeed, through the medium of the *same* nerve, on the stomach, or that these effects should be variously combined with others manifested in the class of spasmodic diseases. Sickness, for example, is sometimes joined with head affection, receiving then the designation of "sick-headach; sometimes with epileptoid seizures, sometimes with attacks of syncope.

The circulation within the medulla oblongata cannot become inordinate without affection of the heart.

Besides the sensations and spasmodic actions about the neck, I have repeatedly heard patients describe similar conditions extending across or around the upper part of the thorax, a state of things which might be mistaken for angina pectoris.

Every symptom attending or constituting sea-sickness points to the medulla oblongata as the part of the nervous system chiefly or primarily affected.

The respiration becomes impaired, a condition which is arrested by drawing a deep sigh. The secretions of the stomach are deranged, and there are acidity and heartburn, and the eructation of flatus. Next the heart becomes feeble in its action, and the countenance turns pallid, or pale and livid.

Then there is a diffused heat of the face and surface; all coverings are thrown off; a clammy perspiration and actual vomiting ensue.

Acid fluid, mucus, bile, are rejected in their turn, and at the last the attacks consist of painful contractions of the diaphragm or hiccup, which sometimes continue for several minutes after the sick ness is over, pointing distinctly, I imagine to the medulla oblongata, as the central nervous organ chiefly affected, as, indeed, it is in every kind of vomiting.

Meantime the cerebrum is unaffected; the intellect is clear; the lower part of the spinal marrow—the lower medulla oblongata—is also unaffected; the sphincters perform their office.

Sea-sickness *resembles* that form of epilepsy which I have noticed as combining nausea and headache, except that there is no affection of the cerebrum; and it partakes, in some measure, of the nature of syncope.

The influence of posture points to irregular circulation in the medulla oblongata, induced by the motion of the vessel, as the *cause* of the series of phenomena in this painful affection.

There is a view of this subject which I approach with great diffidence. A seizure—perhaps a hidden seizure—may take place, and leave a monomaniacal propensity or ruling idea !

Under the influence of this propensity, *Crime* may be committed ! Of such a case the Law, hitherto, equally with Medicine, has taken no cognizance. The crime may be suicidal or homicidal, infanticidal, or sexual, or perhaps directed against truth, honour, or property !

How fearful the consequences of such a state of things might be, I need not say ; but certainly every means should be employed to detect such a hidden seizure in such a case, and especially the temples should be examined, for ecchymosis ; the tongue, for a bitten wound ; the pillow, for marks of foaming at the mouth ; and the linen, for the stains left by some evacuation, whilst the patients should be carefully interrogated, to detect the slightest incoherence or aberration of ideas, or confusion or defect of memory.

Under all circumstances of sudden crime, the possibility of the occurrence of a seizure should be present to the mind ; how much more, if the patient have been epileptic, or if the case be puerperal !

I have already remarked upon the *treatment* of this *Class* of diseases. The principles which should guide us are—

1. To avoid all emotion, or mental effort, or excitement.
2. To preserve the stomach and intestines free from load or oppression.
3. To act on *all* the secretions, especially those of the liver, the kidney, the skin.
4. To combine abundance of walking exercise with early hours ; frictions, with ablutions of the general surface, warmth and dryness of the feet, &c.

It is astonishing to see how much has been effected in many cases apparently the most untoward, by a just combination of, and perseverance in, these remedies.

I beg once more to apologize for the style of these papers. I have not had leisure for composition. They are intended merely to suggest *material for thought and reflection.—London Lancet.*

